# Reduced maximal aerobic capacity after COVID-19 in young adult recruits, Switzerland, May 2020

**DOI:** 10.2807/1560-7917.ES.2020.25.36.2001542

**Published:** 2020-09-10

**Authors:** Giovanni Andrea Gerardo Crameri, Michel Bielecki, Roland Züst, Thomas Werner Buehrer, Zeno Stanga, Jeremy Werner Deuel

**Affiliations:** 1 Swiss Armed Forces, Medical Services, Ittigen, Switzerland; 2 University of Zurich, Institute for Epidemiology, Biostatistics and Prevention Institute, Travel Clinic, Zürich, Switzerland; 3 Federal Office for Civil Protection FOCP, Spiez Laboratory, Spiez, Switzerland; 4 Department of Diabetes, Endocrinology, Nutritional Medicine and Metabolism, Inselspital, Bern University Hospital and University of Bern, Bern, Switzerland; 5 University of Cambridge, Department of Haematology and Wellcome – MRC Cambridge Stem Cell Institute, Jeffrey Cheah Biomedical Centre, Cambridge, United Kingdom

**Keywords:** SARS-CoV-2, physical endurance, lung injury

## Abstract

In March 2020, we observed an outbreak of COVID-19 among a relatively homogenous group of 199 young (median age 21 years; 87% men) Swiss recruits. By comparing physical endurance before and in median 45 days after the outbreak, we found a significant decrease in predicted maximal aerobic capacity in COVID-19 convalescent but not in asymptomatically infected and SARS-CoV-2 naive recruits. This finding might be indicative of lung injury after apparently mild COVID-19 in young adults.

The 2019 coronavirus disease (COVID-19) caused by the severe acute respiratory syndrome coronavirus 2 (SARS-CoV-2) emerged as a pandemic in late 2019, and is ongoing at the time of writing. Long-term sequelae of COVID-19 are still unknown. Pulmonary sequelae impairing physical fitness have been described predominantly for hospitalised patients with COVID-19 [1-3]. Although lung lesions have also been described in asymptomatically infected individuals [3-5], it is yet unclear if these observations correlate with a measurable functional deficit in physical fitness. We performed a well-established and validated physical fitness test [6] before and after an outbreak of COVID-19 among young adult Swiss recruits. By measuring the change in predicted maximal aerobic capacity (VO_2_ max) of not infected, asymptomatically infected and convalescent COVID-19 individuals, we found a decrease in VO_2_ max among COVID-19 convalescent but not among asymptomatically and not infected recruits.

## Description of included recruits and physical fitness tests

We previously described a COVID-19 outbreak at a Swiss Armed Forces Base affecting a cohort of young, predominantly male recruits (173 men, 26 women) with a median age of 21 years (range: 18–27). Throughout military training, recruits who showed symptoms had to immediately report to our clinic where they were swabbed (oronasopharyngeal) and tested for SARS-CoV-2 by reverse transcriptase quantitative PCR (RT-qPCR) for the nsp12 and E-gene. The immune response was measured with commercial enzyme-linked immunosorbent assay kits for IgM, IgG (both from EDI diagnostics, San Diego, United States) and IgA (Euroimmun, Lübeck, Germany). Additionally, we performed cross-sectional sampling on 14 April 2020 to determine the rate of asymptomatic infections [7].

Here we included 199 recruits of two heavily affected companies with available information on SARS-CoV-2 status. Recruits of a further company that was not affected by the outbreak were excluded, since they had a significantly different sex ratio (only men) from the included companies. Participants were grouped by infection status into convalescent recruits with symptomatic confirmed COVID-19 (n = 68), asymptomatic cases with evidence of viral infection by either PCR or serology (n = 77) and a naive group without clinical symptoms or laboratory evidence of SARS-CoV-2 infection (n = 54).

The median time between COVID-19 diagnosis and the fitness test was 45 days (range: 31–58 days), while the baseline test had been performed 3 months before the COVID-19 outbreak at the Armed Forces Base (Table). Paired sport data at baseline and after the COVID-19 outbreak were available for 139 (70%) of the participants. The main reason for missing sport data was medical dispensation, however, the rate of missing data was similar throughout all groups.

**Table:** Characteristics of participating recruits, Switzerland, May 2020

Characteristics	All groups		SARS-CoV-2 naive		Asymptomatically infected		COVID-19 convalescents		p value
Recruits (n, %)	199	100%	54	27%	77	39%	68	34%	NA
Recruits with complete sport data (n, %)	139	70%	36	67%	55	67%	48	71%	NS^a^
Age (years)^b^	20.7 (19.9 to 21.8)		20.8 (20.2 to 22.0)		20.8 (19.9 to 21.9)		20.3 (19.8 to 21.5)		NA
Fraction of female recruits (n, %)	25	13%	6	11%	10	13%	9	13%	NA
BMI (kg/m^2^)^b^	22.7 (21.4 to 24.8)		22.2 (20.5 to 23.9)		23.1 (21.9 to 25.6)		23.7 (21.9 to 25.5)		NA
VO_2_ max at baseline (ml/min/kg)^b^	43.3 (41.8 to 45.1)		43.5 (42.4 to 45.0)		42.8 (41.7 to 44.8)		43.7 (41.5 to 46.0)		NS^c^
VO_2_ max (ml/min/kg)^b^	42.6 (40.8 to 44.5)		42.8 (42.2 to 45.0)		42.9 (40.9 to 44.2)		41.8 (39.9 to 44.6)		NS^c^ (p = 0.06)
Change in VO_2_ max (ml/min/kg)^b^	−0.25 (−2.4 to 1.2)		+ 0.1 (−1.2 to 1.7)		0 (−2.0 to 1.3)		−0.9 (−3.2 to 0.5)		p = 0.005^c^
Fraction of recruits with more than 10% loss in VO_2_ max (n, %)	10	7.2%	0	0.0%	1	1.9%	9	18.8%	p < 0.001^a^
Fraction of recruits with a gain of more than 10% in VO_2_ max (n, %)	9	6.5%	5	13.9%	4	7.6%	0	0%	p = 0.02^a^
Upper extremity strength SSP (m)^b^	5.8 (5.0 to 6.3)		5.8 (5.4 to 6.2)		5.8 (4.9 to 6.5)		6.0 (5.0 to 6.5)		NS^c^
Trunk strength PBT (s)^b^	120 (82 to 180)		130 (115 to 184)		120 (81 to 168)		120 (81 to 149)		NS^c^

COVID-19: coronavirus disease; NA: not applicable; NS: not significant; PBT: prone bridge test; SSP: seated shot put test; SARS-CoV-2: severe acute respiratory syndrome coronavirus 2; VO_2_ max: maximal aerobic capacity.

^a^ Fisher’s exact test.

^b^ The median and 25%–75% percentiles in brackets.

^c^ Student’s t-test comparing COVID-19 patients to the other two groups.

Aerobic endurance, trunk muscle, and upper extremity strength are measured by a progressive endurance run (PER), prone bridge test (PBT) and seated shot put test (SSP), respectively. Using the final running velocity of the PER, predicted maximal aerobic capacity (VO_2_ max) can be calculated [8,9]. Since the local outbreak occurred in-between the two sets of tests, we were able to assess physical fitness before and after infection with SARS-CoV-2. Statistical significance testing for differences of scalar variables (e.g. VO_2_ max) between the groups were performed with Student’s t-test and differences in the contingency tables were tested with Fisher’s exact test. p values smaller than 0.05 were considered as statistically significant.

## Decreased maximal aerobic capacity in COVID-19 convalescent recruits

We observed a statistically significant decrease in VO_2_ max among COVID-19 convalescents compared with naive and asymptomatically infected recruits (Figure). While the median VO_2_ max stayed unchanged for most recruits, a significant decrease in VO_2_ max of −0.9 ml/min/kg was observed in COVID-19 convalescents if compared with their individual baseline measurements before the local outbreak (Figure). Not only the difference but also the absolute VO_2_ max was lower in convalescent as compared with naive recruits (p = 0.02, data not shown), although significance was lost if COVID-19 convalescents were compared with the combined group of asymptomatic and naive recruits (p = 0.06, Table).

**Figure:**
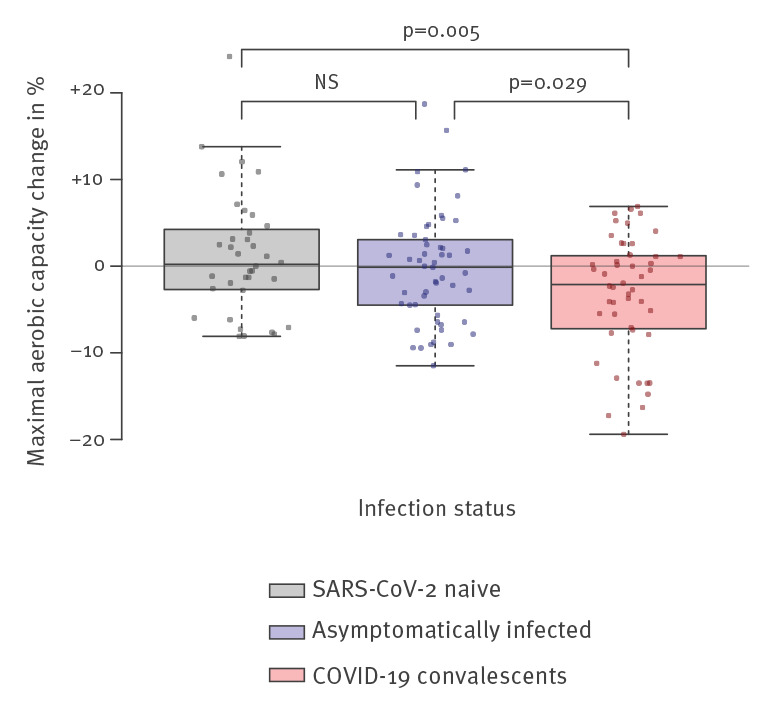
Difference in predicted maximal aerobic capacity before and after COVID-19 outbreak by infection status among recruits, Switzerland, May 2020

COVID-19: coronavirus disease; NS: not significant; SARS-CoV-2: severe acute respiratory syndrome coronavirus 2.

p values were calculated by Student’s t-test.

Neither of the strength tests (PBT and SSP) differed significantly between the three groups (Table).

During military training, fitness is expected to generally increase. We observed an increase in VO_2_ max of more than 10% in nearly 14% of the naive recruits and nearly 8% in the asymptomatic group, while none of the COVID-19 convalescent recruits had an increase of more than 10%. On the contrary, 19% of the COVID-19 convalescents had a decrease of more than 10% in VO_2_ max, while none of the naive recruits showed such a decrease. While the overall effect of COVID-19 on VO_2_ max might be subtle, a significant subset of patients seem to have lost more than 10% of their initial aerobic capacity.

These findings indicate a specific decrease in aerobic capacity affecting COVID-19 convalescent recruits compared with naive as well as asymptomatically infected ones. VO_2_ max before the COVID-19 outbreak was very similar between the groups, excluding a selection bias for preexisting lower scores.

## Ethical statement

All procedures involving human participants were conducted in accordance with the ethical standards of the Declaration of Helsinki, as revised in 2013. The study protocol was approved by the ethical committee of the Canton of Ticino, Switzerland (BASEC-ID 2020–00623 CE 3609). Written informed consent of all participants was obtained after oral explanations provided in their native language (German, French or Italian).

## Discussion and conclusions

To our knowledge, changes in endurance or strength following SARS-CoV-2 infection, symptomatic or not, have not been described until now. We showed reduced aerobic capacity in young adult recruits 1 to 2 months after symptomatic COVID-19 while physical strength was unaffected. Ca 19% of COVID-19 convalescent recruits showed a decrease of VO_2_ max of more than 10% as compared with baseline before infection. Although our data do not explain the pathophysiology behind these findings, reduced VO_2_ max is a hallmark of interstitial lung disease [10]. SARS-CoV-2 infection has been described to induce lung damage, even in asymptomatic cases [4]. This indicates the importance of further long-term follow-up studies to assess the extent and duration of the sequelae, as well as of infection prevention to avoid these long-term consequences.

Physical deconditioning or demotivation may explain impaired fitness or compliance, respectively. The COVID-19 outbreak had a mental and physical impact on military personnel: stringent physical distancing measures, quarantine and isolation restricted possibilities for physical activity, and lowered morale. However, we would expect deconditioning and lowered test adherence due to demotivation to affect the results for both aerobic capacity and physical strength similarly [11], which was not the case with our observations.

Other than the described endurance run, we could neither conduct more specific tests (such as spirometry) nor perform serial imaging to identify our results’ pathophysiology. The cohort has meanwhile been dissolved due to the end of military training, thus such studies are difficult to perform in our study group. Since we studied a relatively homogeneous cohort of young, otherwise healthy, and predominantly male adults, our findings might not be applicable to other population groups.

Our observations were made within 1 to 2 months after the diagnosis of COVID-19 and follow-up studies should be conducted to determine whether the reduction in VO_2_ max is reversible. With ca 44,900 confirmed COVID-19 cases in Switzerland [12] as well as over 2.4 million in the European Union and Economic Area and the United Kingdom, respectively [13] and still increasing case numbers, evaluating possible long-term consequences of COVID-19 is becoming more important by the day.
